# Supplementary material for: In Vitro and In Silico Analysis of PTP1B Inhibitors from Cleistocalyx operculatus Leaves and Their Effect on Glucose Uptake
Source: Nutrients. 2024 Aug 24;16(17):2839. doi: 10.3390/nu16172839 (PMC11397035; doi:10.3390/nu16172839)
Supplement: Supplementary file 1 [file nutrients-16-02839-s001.zip › nutrients-3162589-supplementary.pdf]

Supporting Information

In Vitro and In Silico Analysis of PTP1B Inhibitors from *Cleistocalyx operculatus* Leaves and Their Effect on Glucose Uptake

Jorge-Eduardo Ponce-Zea [1,†], Byeol Ryu [1,†], Ju-Yong Lee [1], Eun-Jin Park [1], Van-Hieu Mai [1], Thi-Phuong Doan [1], Hee Ju Lee [2], and Won Keun Oh [1,*]

[1] *Research Institute of Pharmaceutical Sciences, College of Pharmacy, Seoul National University, Seoul 08826, Republic of Korea*

[2] *Natural Product Informatics Research Center, Korea Institute of Science and Technology, Gangneung, 25451, Republic of Korea*

[*] To whom correspondence should be address. Tel & Fax: +82-02-880-7872. E-mail: wkoh1@snu.ac.kr.

[†] Jorge-Eduardo Ponce-Zea and [†] Byeol Ryu contributed equally to this work, they are the co-first authors.

List of Supporting information

Figure S1. HR-ESI-MS spectrum of compound **1** 4

Figure S2. IR spectrum of compound **1** 4

Figure S3. ^{1}H NMR spectrum of compound **1** in pyridine-d_5 5

Figure S4. ^{13}C NMR spectrum of compound **1** in pyridine-d_5 5

Figure S5. HSQC spectrum of compound **1** in pyridine-d_5 6

Figure S6. HMBC spectrum of compound **1** in pyridine-d_5 6

Figure S7. NOESY spectrum of compound **1** in pyridine-d_5 7

Figure S8. HR-ESI-MS spectrum of compound **2** 8

Figure S9. IR spectrum of compound **2** 8

Figure S10. ^{1}H NMR spectrum of compound **2** in pyridine-d_5 9

Figure S11. ^{13}C NMR spectrum of compound **2** in pyridine-d_5 9

Figure S12. HSQC spectrum of compound **2** in pyridine-d_5 10

Figure S13. HMBC spectrum of compound **2** in pyridine-d_5 10

Figure S14. NOESY spectrum of compound **2** in pyridine-d_5 11

Figure S15. HR-ESI-MS spectrum of compound **3** 12

Figure S16. IR spectrum of compound **3** 12

Figure S17. ^{1}H NMR spectrum of compound **3** in pyridine-d_5 13

Figure S18. ^{13}C NMR spectrum of compound **3** in pyridine-d_5 13

Figure S19. HSQC spectrum of compound **3** in pyridine-d_5 14

Figure S20. HMBC spectrum of compound **3** in pyridine-d_5 14

Figure S21. NOESY spectrum of compound **3** in pyridine-d_5 15

Figure S22. PTP1B inhibition activity screening of compounds **1** – **17** 16

Figure S23. PTP1B inhibition activity of compounds **4**, **5**, and **6** 17

Figure S24. PTP1B inhibition activity of compounds **9**, **10**, and **11** 17

Figure S25. PTP1B inhibition activity of compounds **13**, **14**, and **15** 18

Figure S26. PTP1B inhibition activity of compounds **16** and **17** 18

Figure S27. Overlapped docked structures of compounds 6 (green), 9 (orange), 17 (purple), and positive control (yellow, PDB:1T49 ligand) in the allosteric binding site of PTP1B (PDB:1T49)....................19

Figure S28. Glucose uptake and PTP1B inhibition pattern among isolated compounds. Non-parametric Spearman correlation, $R = 0.51$, $p = 0.03$, p-value two-tailed, confidence interval 95%. PTP1B IC_{50} and glucose uptake values were ranked on Excel to perform statistical correlation. Graph and statistical analysis were carried on GraphPad Prism 10..19

Figure S29. UPLC-qTOF MS/MS chromatogram of the hot water extract at 40 °C (upper chromatogram) and the 50% EtOH extract (lower chromatogram). The content of two extracts is similar in the polar to medium polarity region..20

Table S1. ADMET profile of isolated compounds predicted by QSAR regression models.................21

Elemental Composition Report **Page 1**

Single Mass Analysis
Tolerance = 10.0 PPM / DBE: min = -1.5, max = 50.0
Element prediction: Off
Number of isotope peaks used for i-FIT = 3

Monoisotopic Mass, Even Electron Ions
139 formula(e) evaluated with 2 results within limits (up to 50 closest results for each mass)
Elements Used:
C: 0-500 H: 0-1000 O: 0-200
211123_CO1_neg
Compound 1 3384 (12.889) 1: TOF MS ES-
4.13e+004

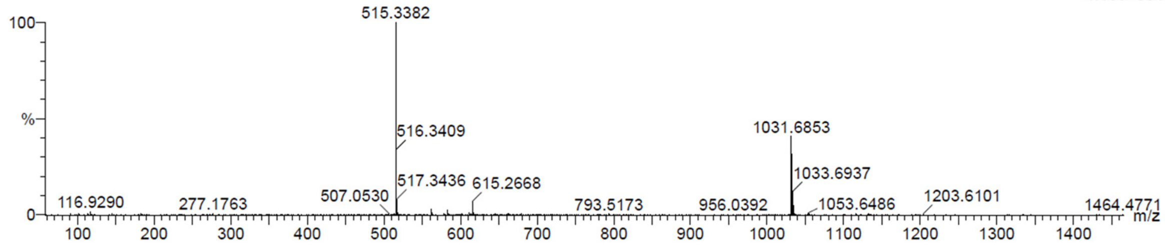

Minimum:				-1.5				
Maximum:		5.0	10.0	50.0				
Mass	Calc. Mass	mDa	PPM	DBE	i-FIT	Norm	Conf(%)	Formula
515.3382	515.3373	0.9	1.7	8.5	126.6	0.001	99.92	C31 H47 O6
	515.3431	-4.9	-9.5	-0.5	133.7	7.102	0.08	C24 H51 O11

Figure S1. HRESI(-)MS spectrum of compound **1**

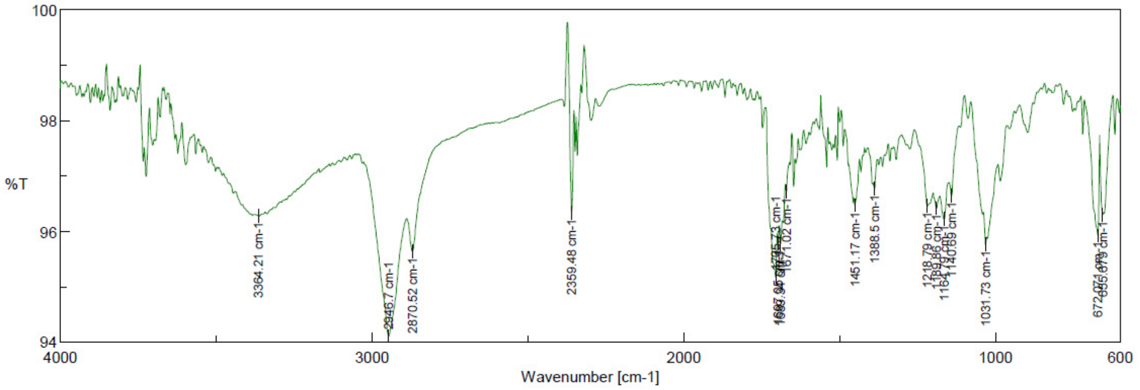

[Comment]		[Measurement Information]	
Sample Name		Model Name	FT/IR-4200typeA
Comment		Serial Number	B038361018
User			
Division		Light Source	Standard
Company	공동기기실	Detector	TGS
		Accumulation	Auto (23)
[Data Information]		Resolution	4 cm-1
Creation Date	2017-10-25 오전 10:53	Zero Filling	On
Data array type	Linear data array	Apodization	Cosine
Horizontal	Wavenumber [cm-1]	Gain	Auto (2)
Vertical	%T	Aperture	Auto (7.1 mm)
Start	599.753 cm-1	Scanning Speed	Auto (2 mm/sec)
End	4000.6 cm-1	Filter	Auto (30000 Hz)
Data pitch	0.964233 cm-1		
Data points	3528		

Figure S2. IR spectrum of compound **1**

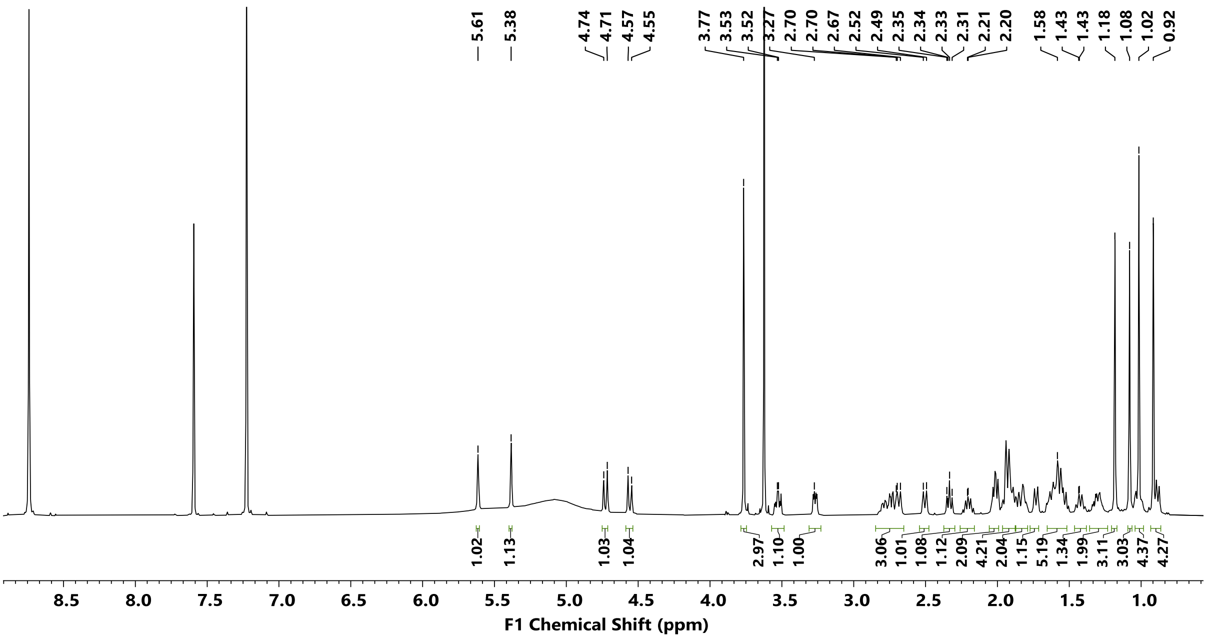

Figure S3. ^{1}H NMR spectrum of compound **1** in pyridine-d_5

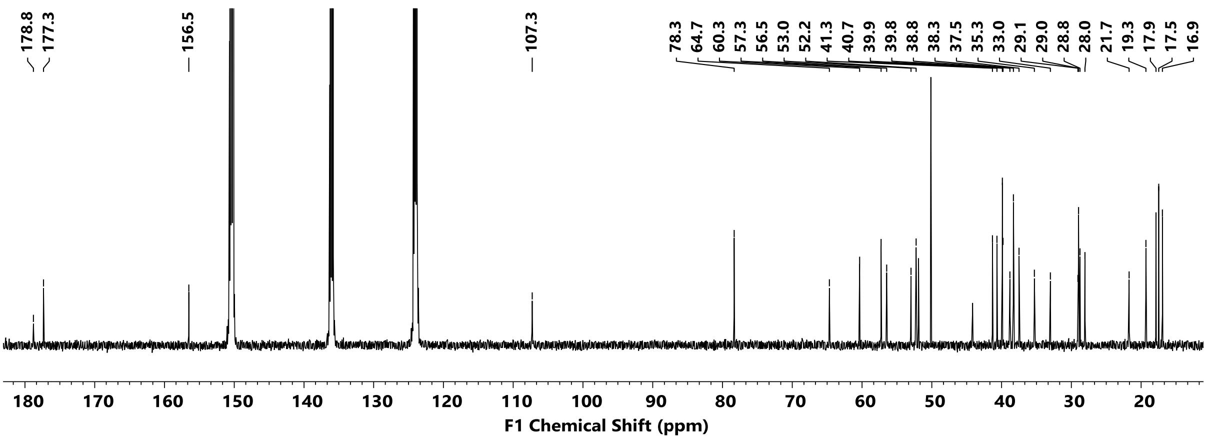

Figure S4. ^{13}C NMR spectrum of compound **1** in pyridine-d_5

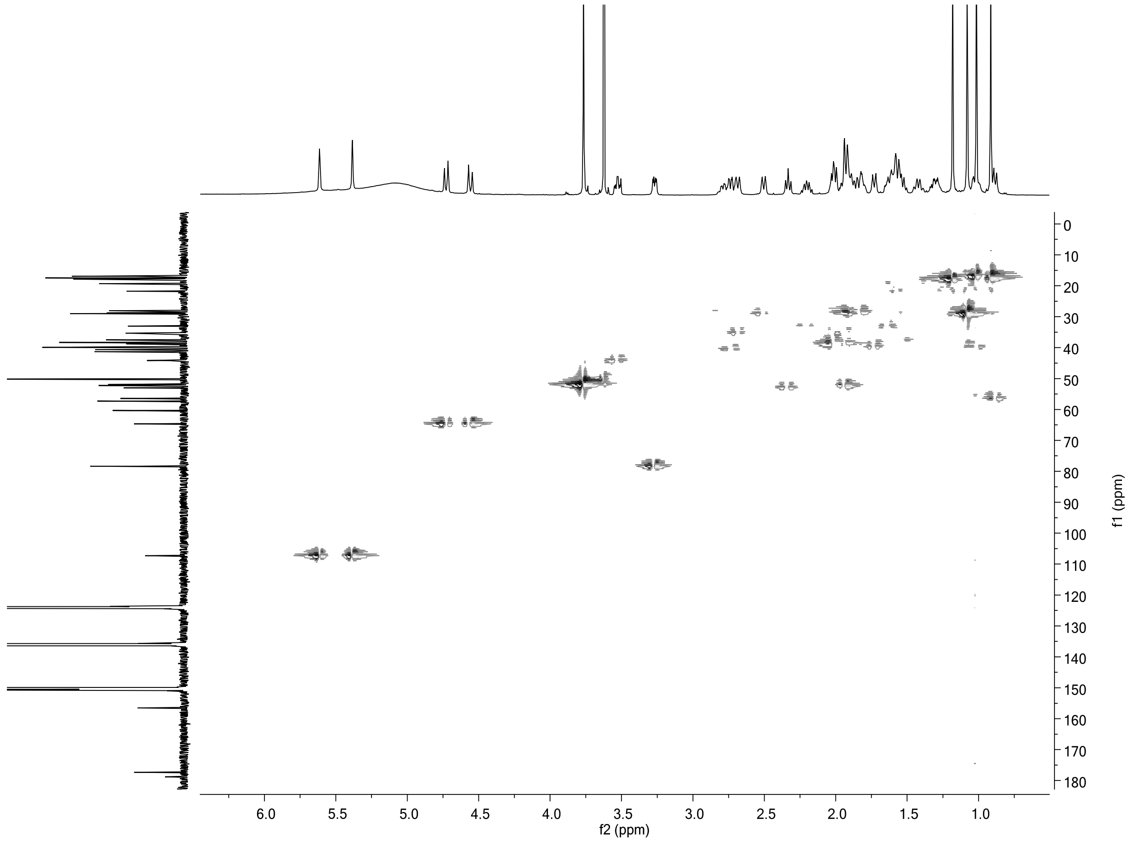

Figure S5. HSQC spectrum of compound **1** in pyridine-*d*$_5$

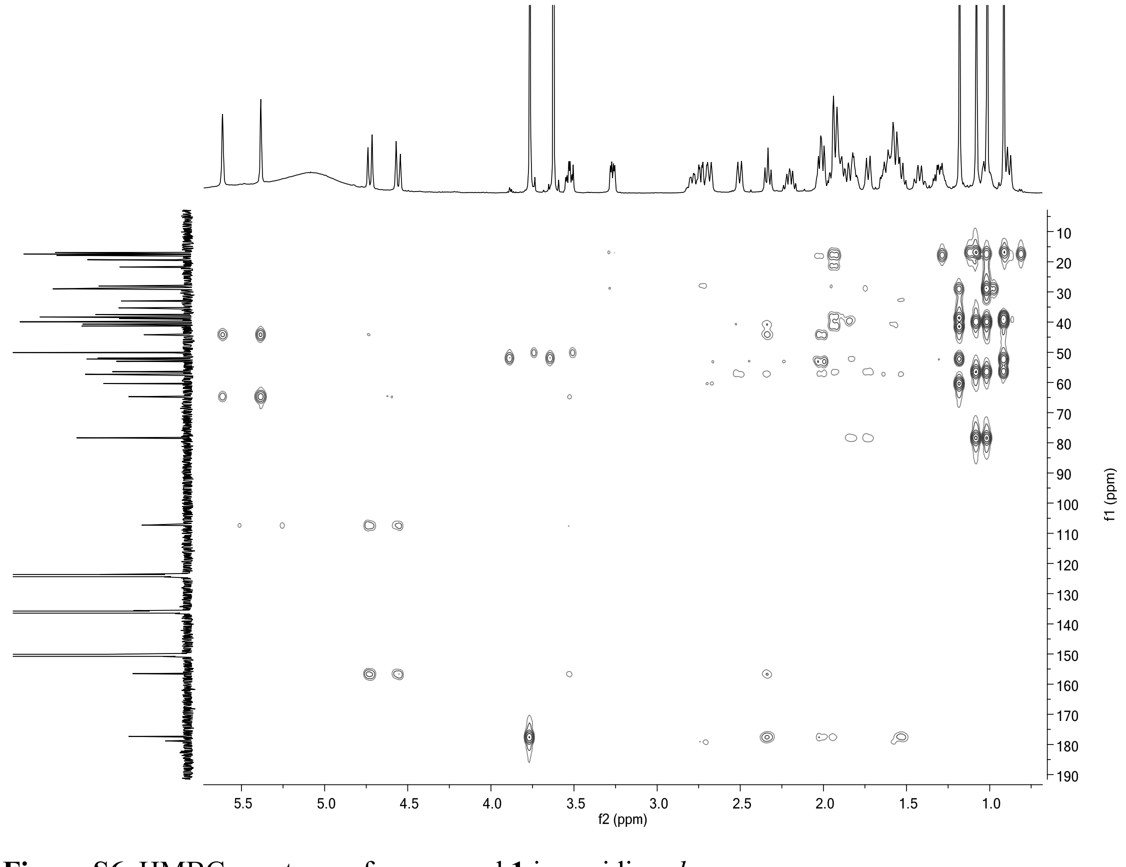

Figure S6. HMBC spectrum of compound **1** in pyridine-*d*$_5$

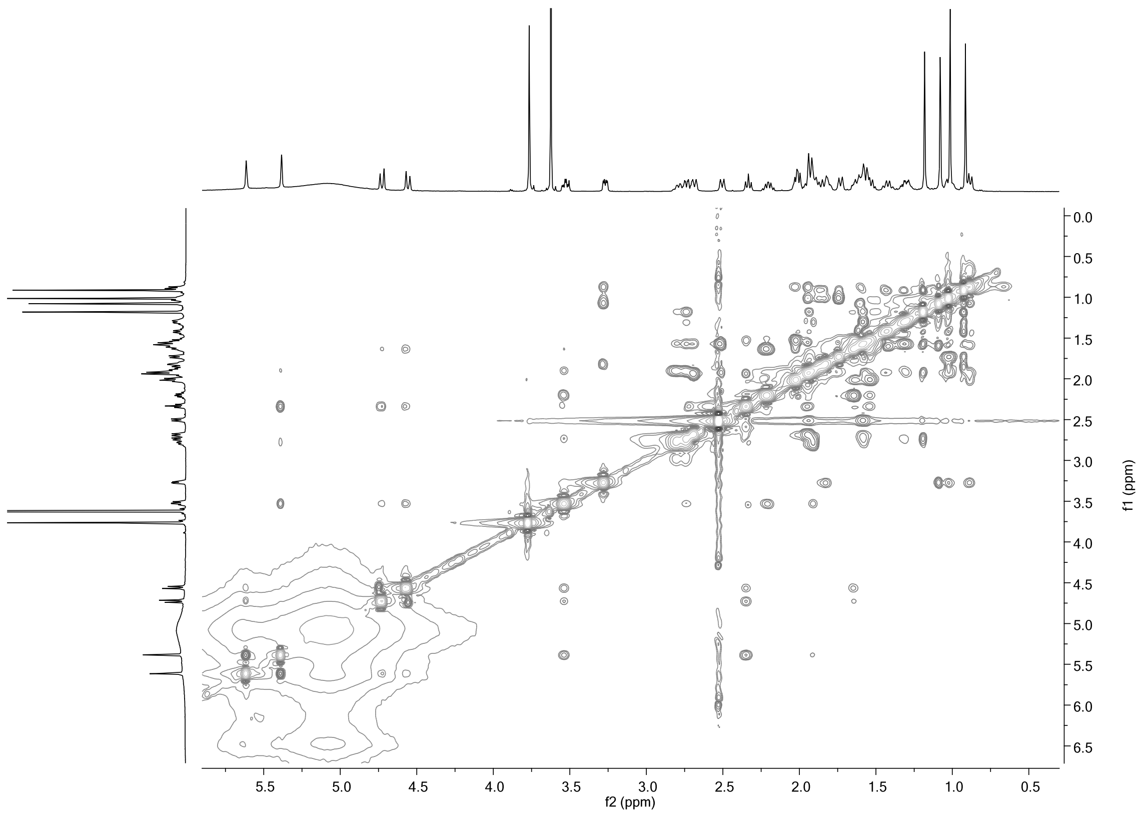

Figure S7. NOESY spectrum of compound **1** in pyridine-d_5

Single Mass Analysis
Tolerance = 10.0 PPM / DBE: min = -1.5, max = 50.0
Element prediction: Off
Number of isotope peaks used for i-FIT = 3

Monoisotopic Mass, Even Electron Ions
139 formula(e) evaluated with 1 results within limits (up to 50 closest results for each mass)
Elements Used:
C: 0-500 H: 0-1000 O: 0-200
211123_CO3_neg
Compound 3 3746 (14.639) 1: TOF MS ES- 2.98e+003

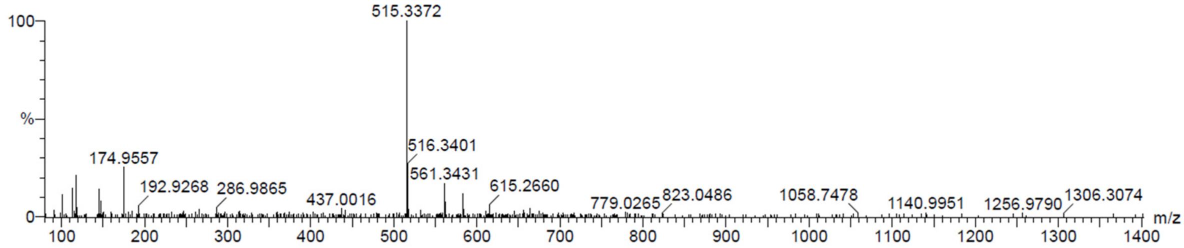

Minimum:				-1.5				
Maximum:		5.0	10.0	50.0				
Mass	Calc. Mass	mDa	PPM	DBE	i-FIT	Norm	Conf(%)	Formula
515.3372	515.3373	-0.1	-0.2	8.5	22.1	n/a	n/a	C31 H47 O6

Figure S8. HRESI(-)MS spectrum of compound **2**

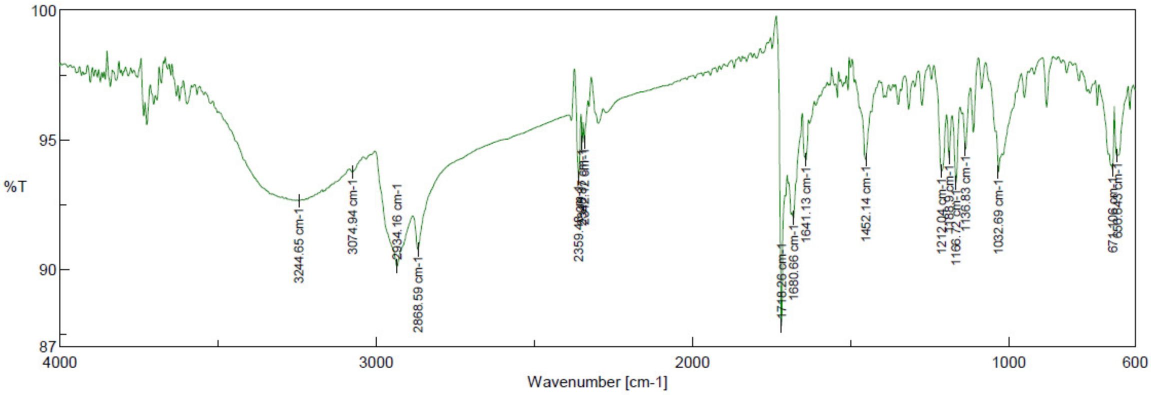

[Comment]		[Measurement Information]		
Sample Name		Model Name	FT/IR-4200typeA	co 3.jws
Comment		Serial Number	B038361018	
User				
Division		Light Source	Standard	
Company	공동기기실	Detector	TGS	
		Accumulation	Auto (26)	
[Data Information]		Resolution	4 cm-1	
Creation Date	2017-10-25 오전 11:00	Zero Filling	On	
Data array type	Linear data array	Apodization	Cosine	
Horizontal	Wavenumber [cm-1]	Gain	Auto (2)	
Vertical	%T	Aperture	Auto (7.1 mm)	
Start	599.753 cm-1	Scanning Speed	Auto (2 mm/sec)	
End	4000.6 cm-1	Filter	Auto (30000 Hz)	
Data pitch	0.964233 cm-1			
Data points	3528			

Figure S9. IR spectrum of compound **2**

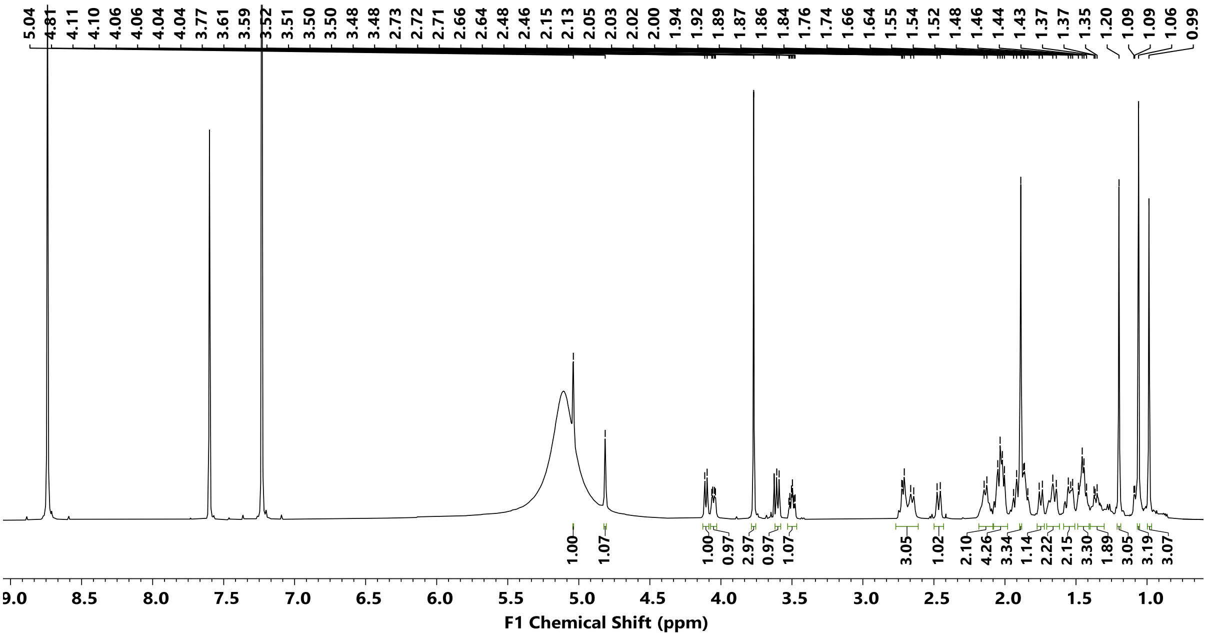

Figure S10. ^{1}H NMR spectrum of compound **2** in pyridine-d_5

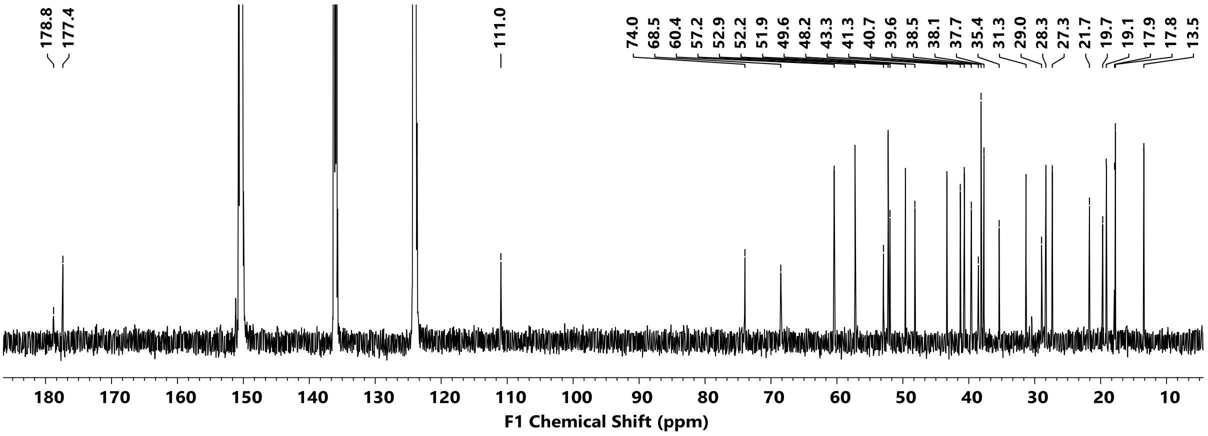

Figure S11. ^{13}C NMR spectrum of compound **2** in pyridine-d_5

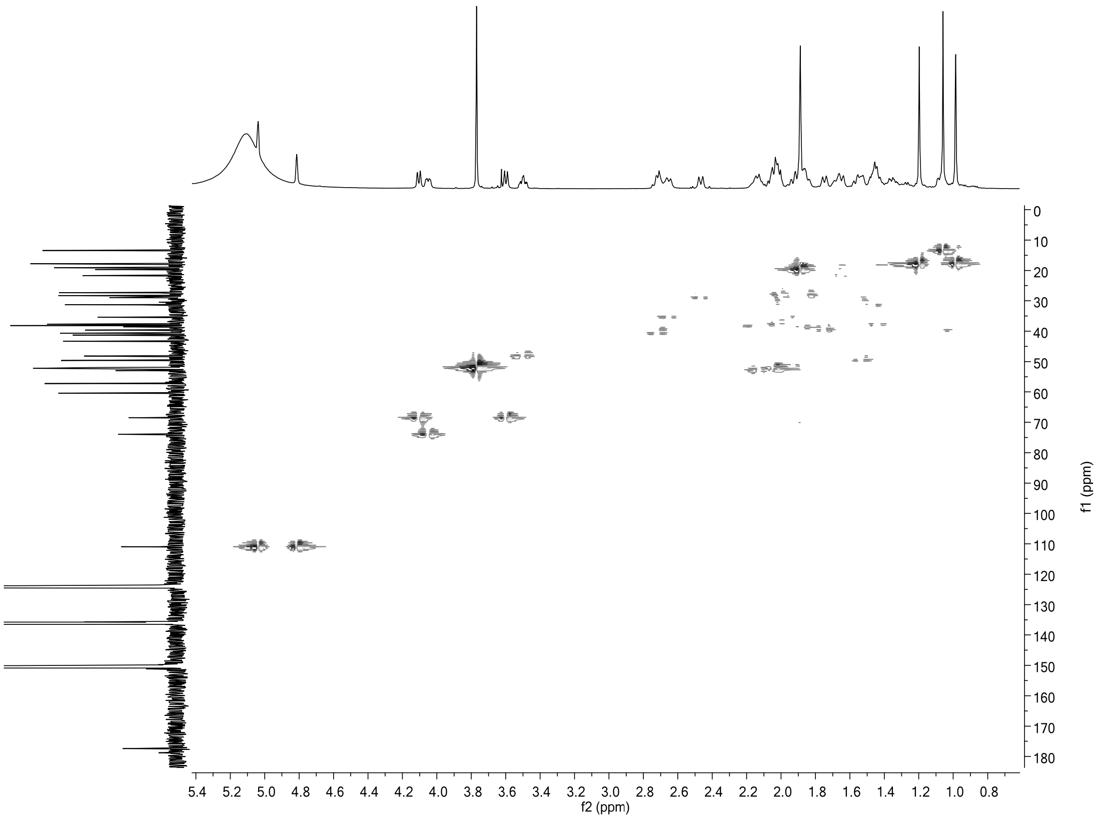

Figure S12. HSQC spectrum of compound **2** in pyridine-d_5

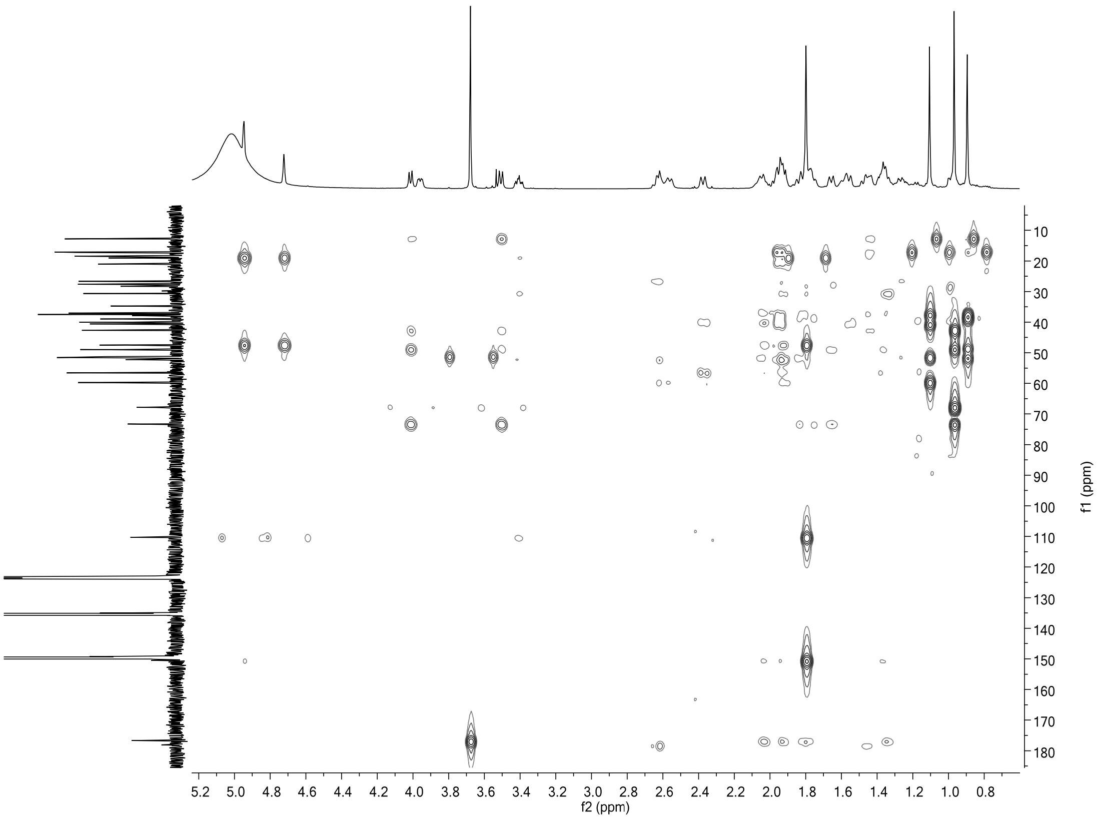

Figure S13. HMBC spectrum of compound **2** in pyridine-d_5

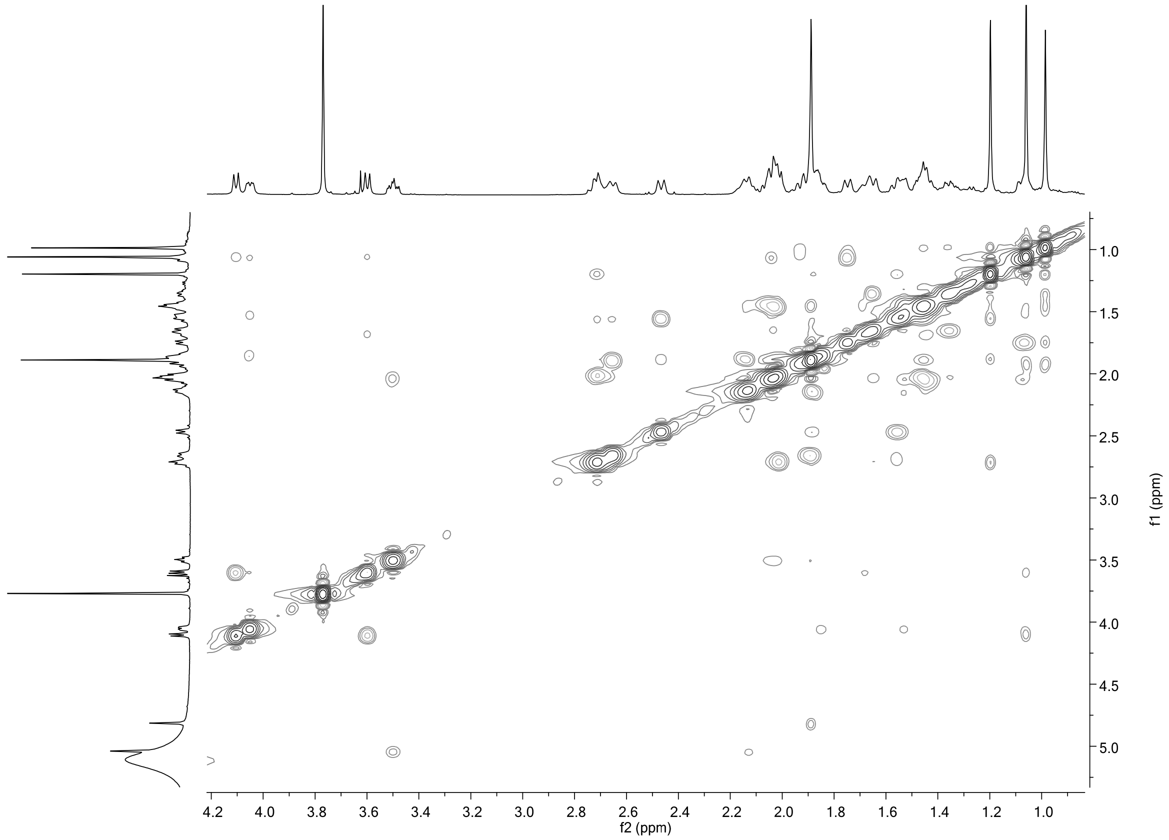

Figure S14. NOESY spectrum of compound **2** in pyridine-d_5

Elemental Composition Report

Page 1

Single Mass Analysis

Tolerance = 10.0 PPM / DBE: min = -1.5, max = 50.0
Element prediction: Off
Number of isotope peaks used for i-FIT = 3

Monoisotopic Mass, Even Electron Ions
132 formula(e) evaluated with 1 results within limits (up to 50 closest results for each mass)
Elements Used:
C: 0-500 H: 0-1000 O: 0-200
211123_CO2_neg
Compound 2 3182 (11.794)

1: TOF MS ES-
3.55e+004

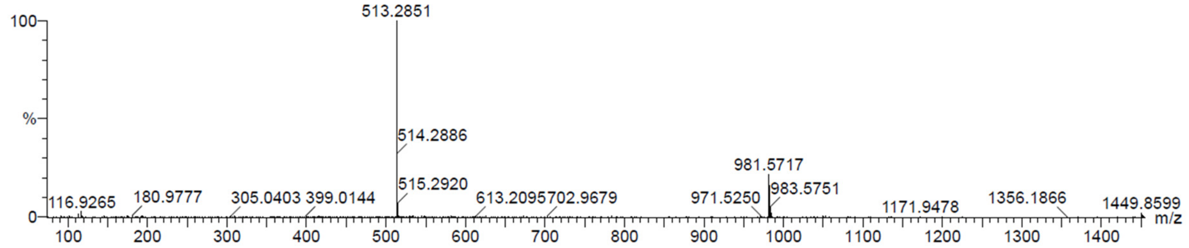

Minimum:				-1.5				
Maximum:		5.0	10.0	50.0				

Mass	Calc. Mass	mDa	PPM	DBE	i-FIT	Norm	Conf(%)	Formula
513.2851	513.2852	-0.1	-0.2	10.5	129.4	n/a	n/a	C30 H41 O7

Figure S15. HRESI(-)MS spectrum of compound **3**

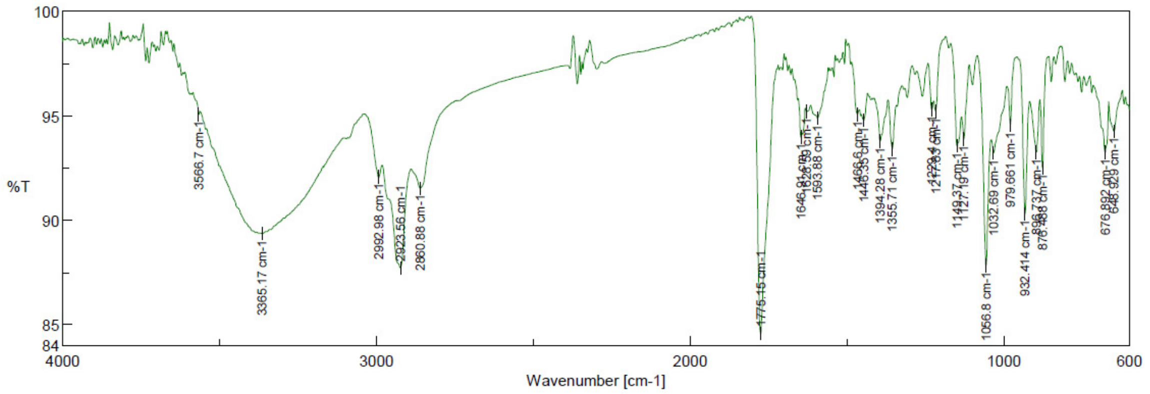

[Comment]		[Measurement Information]	
Sample Name		Model Name	FT/IR-4200typeA
Comment		Serial Number	B038361018
User			
Division		Light Source	Standard
Company	공동기기실	Detector	TGS
		Accumulation	Auto (27)
[Data Information]		Resolution	4 cm-1
Creation Date	2017-10-25 오전 10:57	Zero Filling	On
Data array type	Linear data array	Apodization	Cosine
Horizontal	Wavenumber [cm-1]	Gain	Auto (2)
Vertical	%T	Aperture	Auto (7.1 mm)
Start	599.753 cm-1	Scanning Speed	Auto (2 mm/sec)
End	4000.6 cm-1	Filter	Auto (30000 Hz)
Data pitch	0.964233 cm-1		
Data points	3528		

co 2.jws

Figure S16. IR spectrum of compound **3**

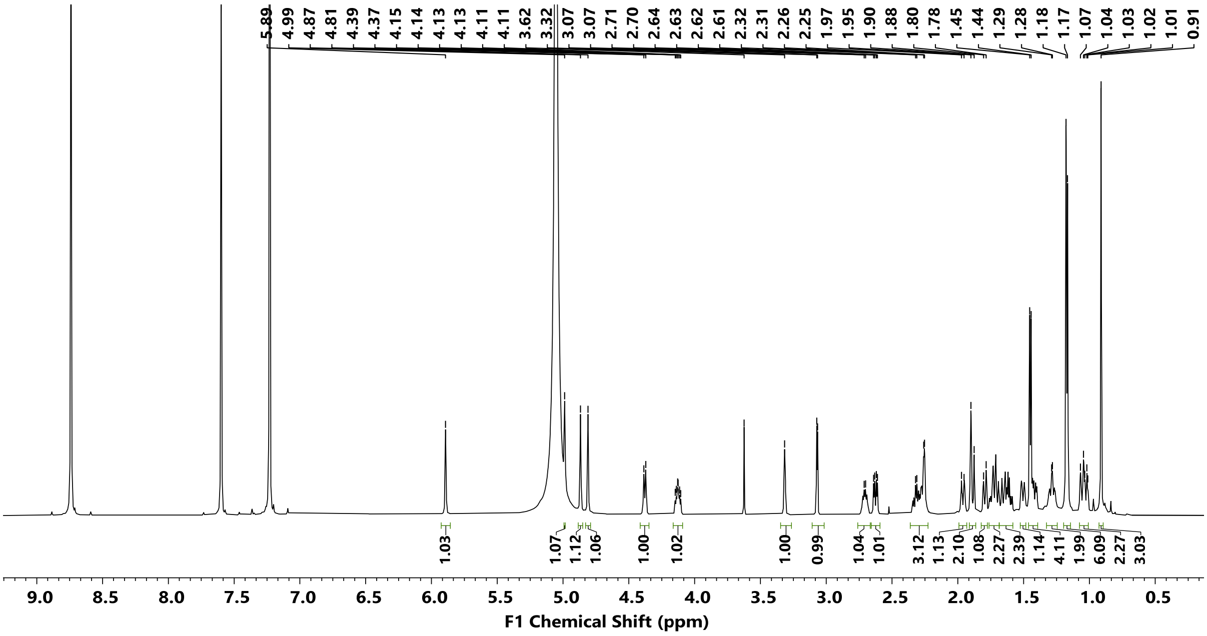

Figure S17. ^{1}H NMR spectrum of compound **3** in pyridine-d_5

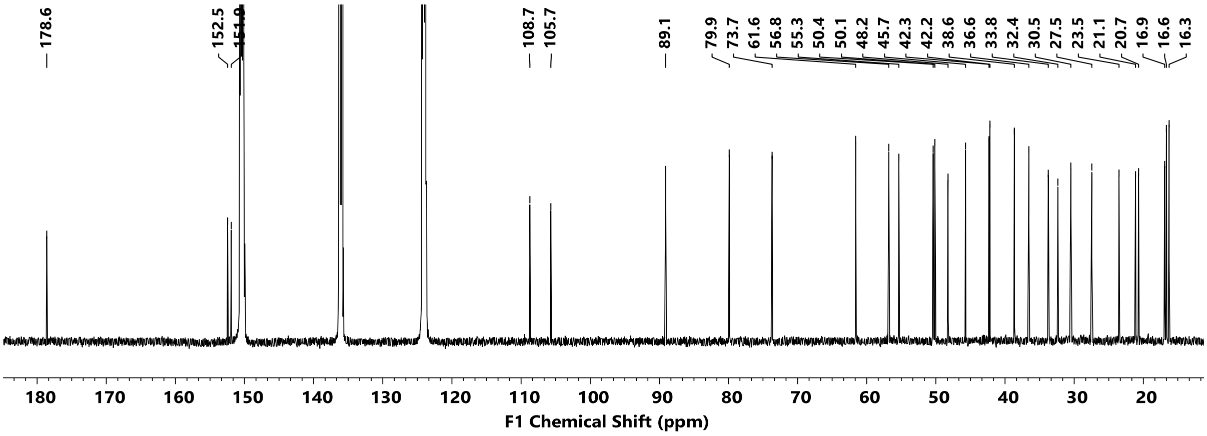

Figure S18. ^{13}C NMR spectrum of compound **3** in pyridine-d_5

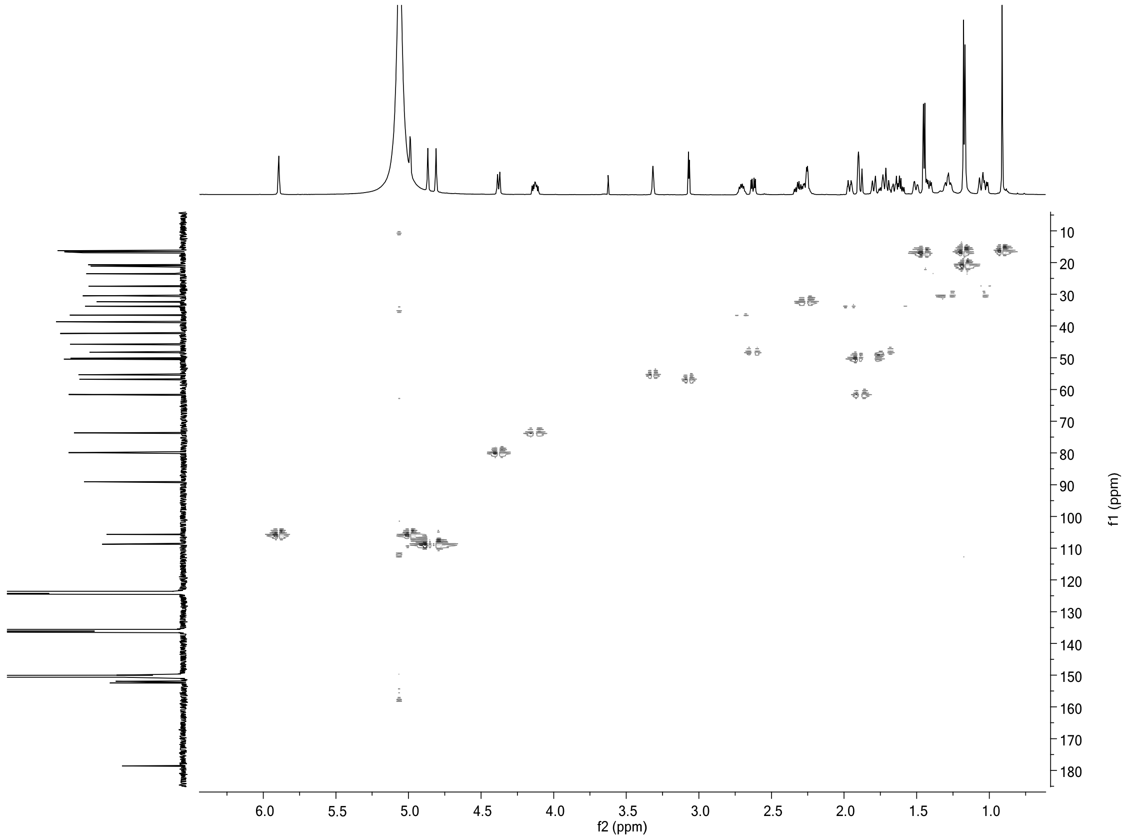

Figure S19. HSQC spectrum of compound **3** in pyridine-d_5

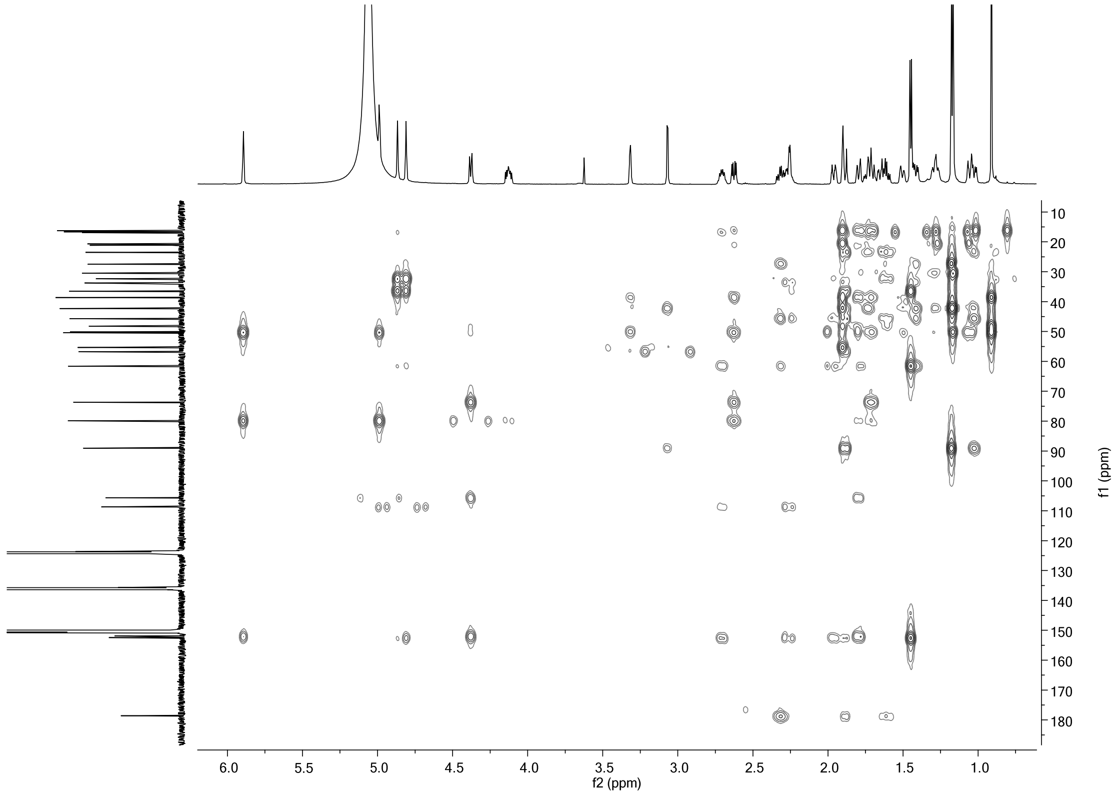

Figure S20. HMBC spectrum of compound **3** in pyridine-d_5

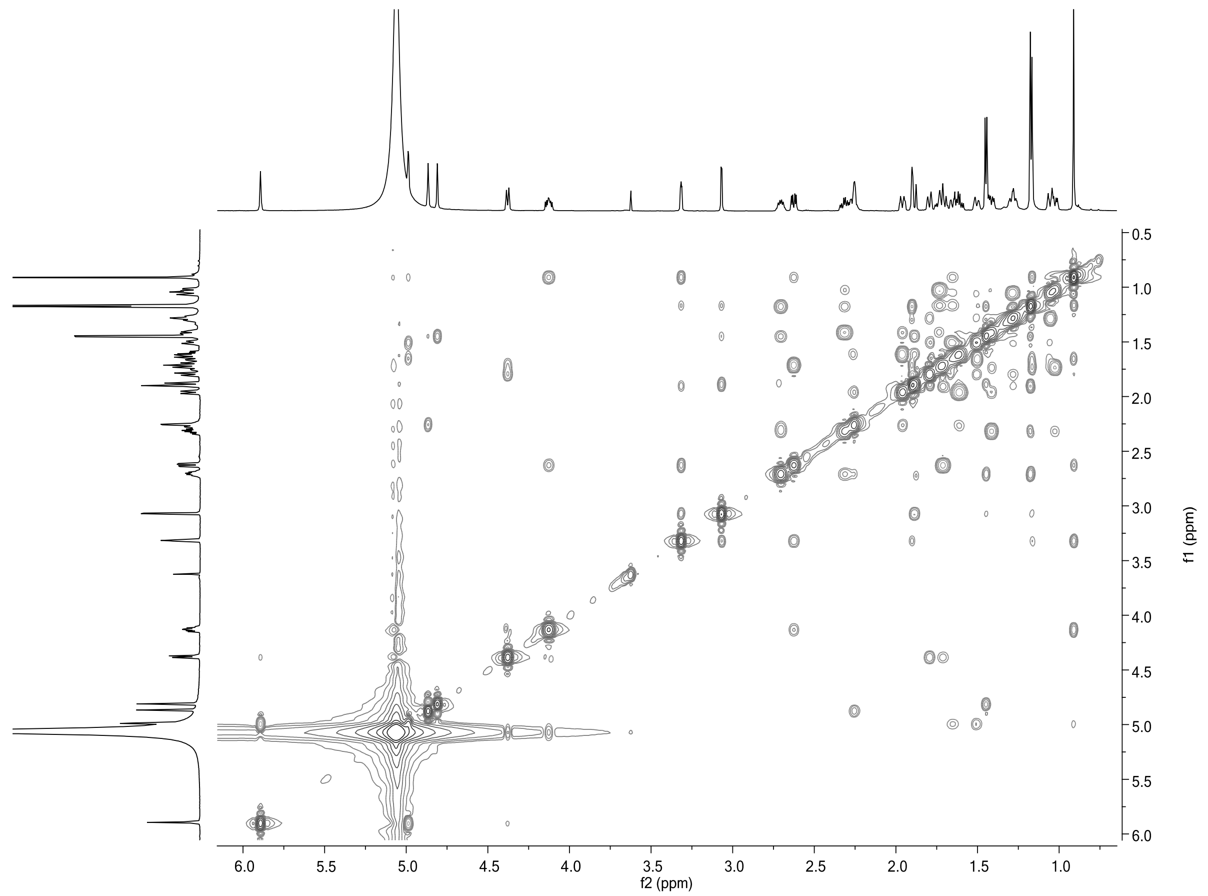

Figure S21. NOESY spectrum of compound **3** in pyridine-d_5

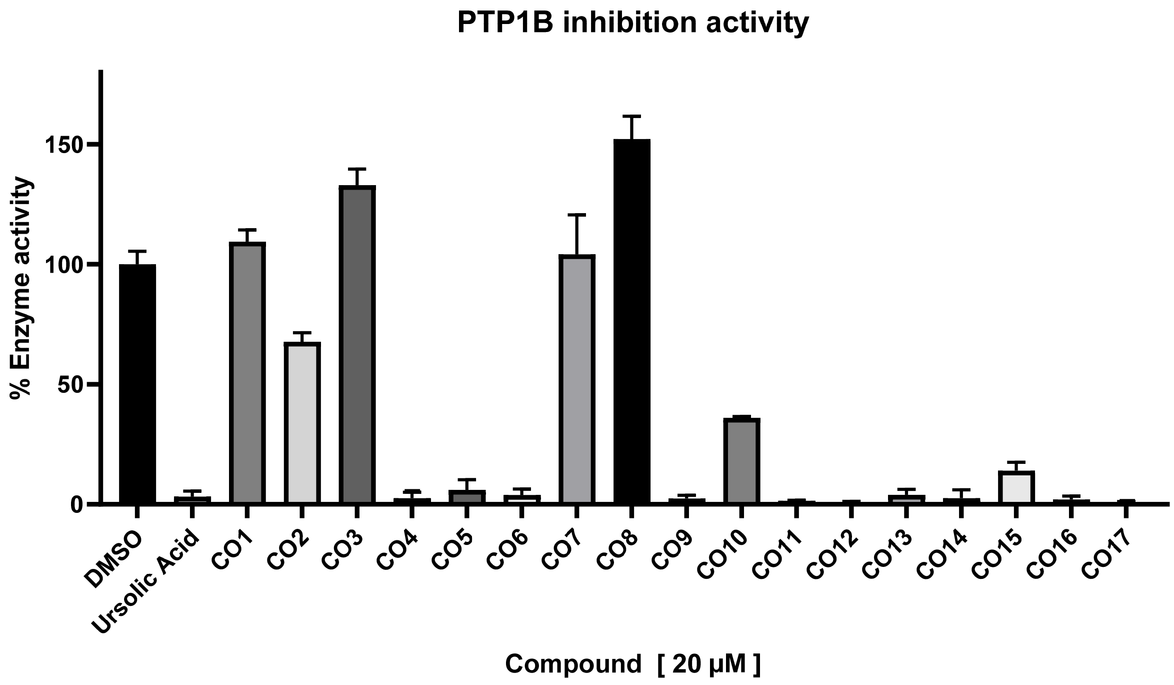

Figure S22. Assessment of the inhibitory effects of compounds **1 – 17** (20 μM) on PTP1B

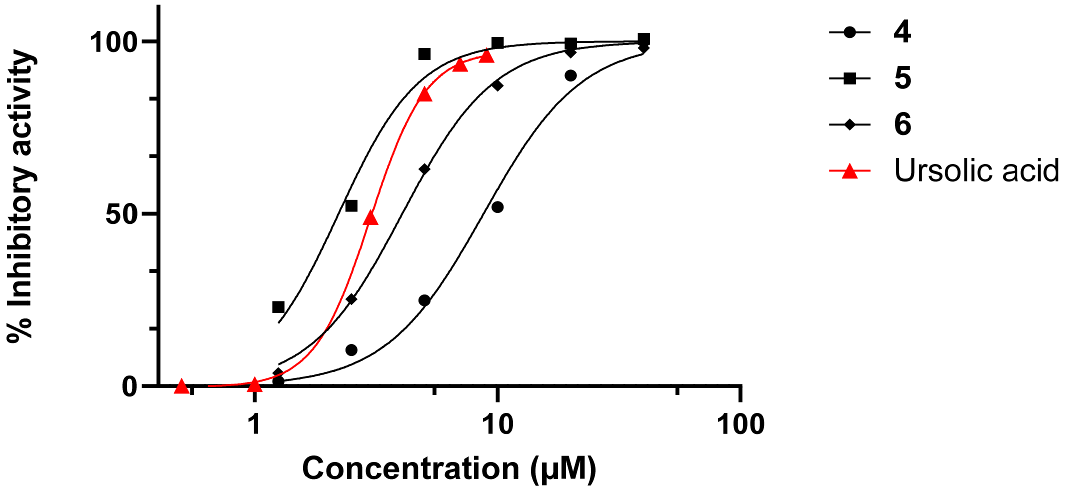

Figure S23. PTP1B inhibition activity of **4**, **5** and **6**.

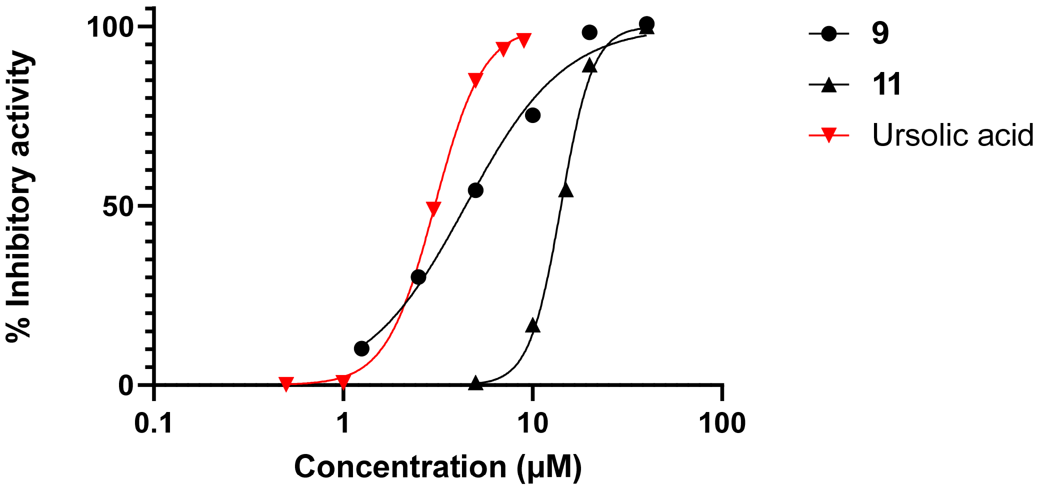

Figure S24. PTP1B inhibition activity of **9** and **11**

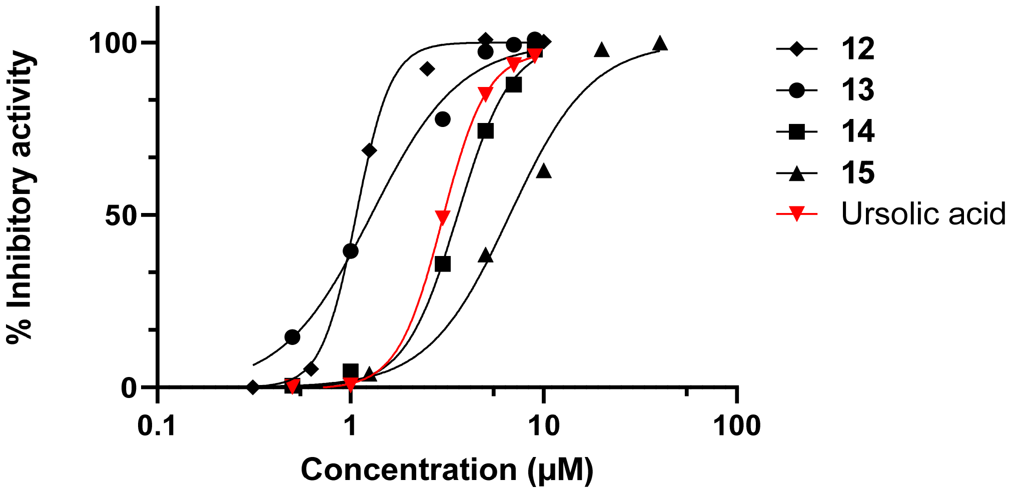

Figure S25. PTP1B inhibition activity of **13, 14** and **15**

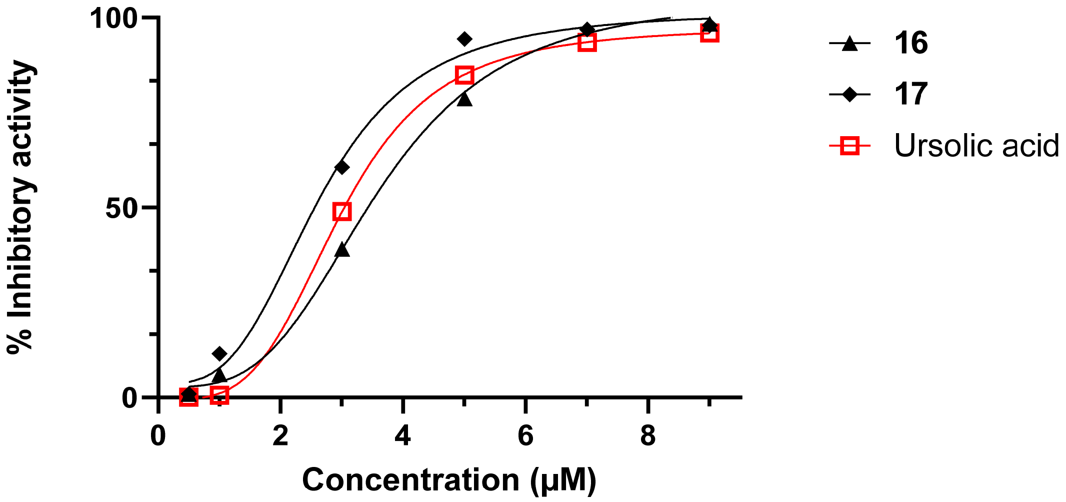

Figure S26. PTP1B inhibition activity of **16** and **17**

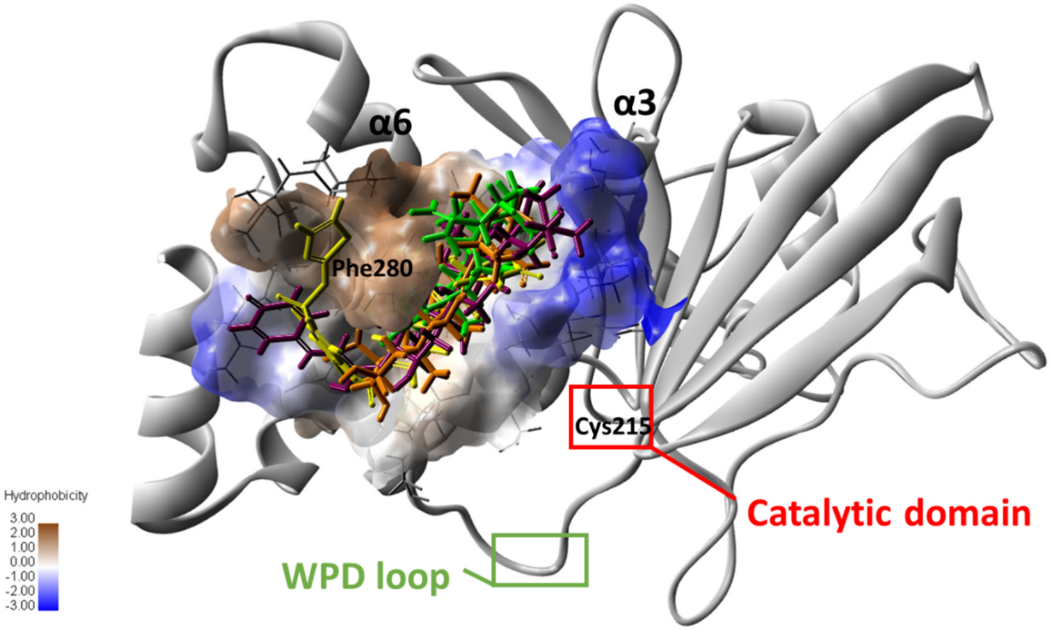

Figure S27. Overlapped docked structures of compounds **6** (green), **9** (orange), **17** (purple), and positive control (yellow, PDB:1T49 ligand) in the allosteric binding site of PTP1B (PDB:1T49).

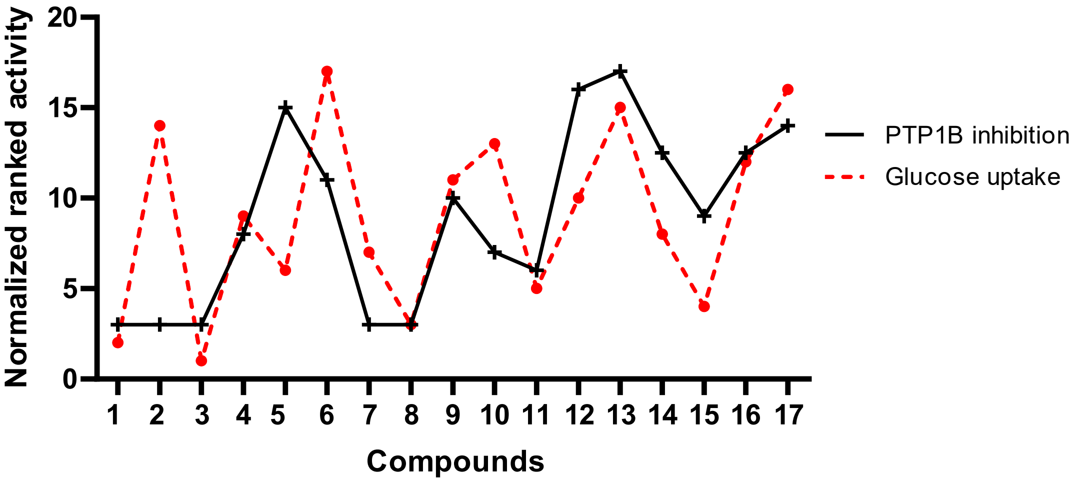

Figure S28. Glucose uptake and PTP1B inhibition pattern among isolated compounds. Non-parametric Spearman correlation, $R = 0.51$, $p = 0.03$, p-value two-tailed, confidence interval 95%. PTP1B IC_{50} and glucose uptake values were ranked on Excel to perform statistical correlation. Graph and statistical analysis were carried on GraphPad Prism 10.

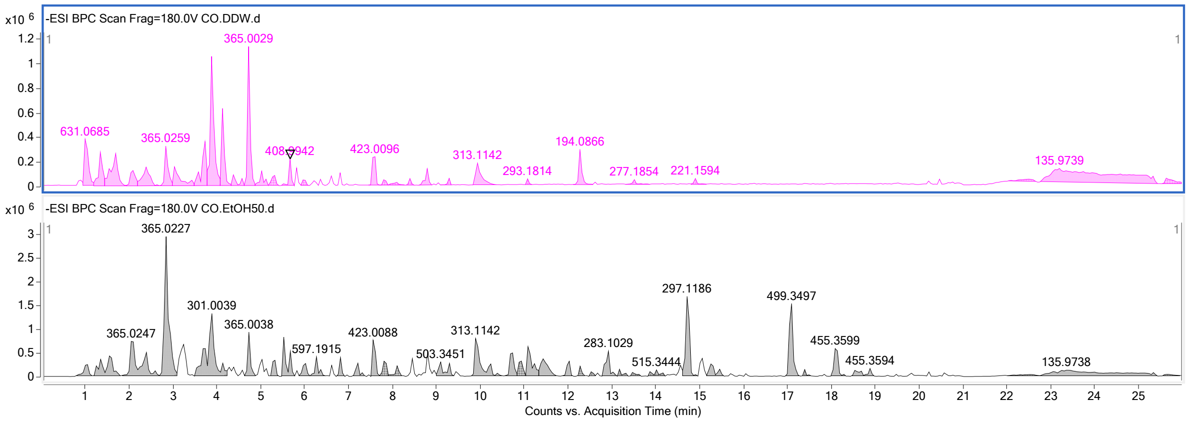

Figure S29. LC-MS chromatogram (negative ionization mode) of hot water extract 40 °C(upper chromatogram) and 50% EtOH extract (down chromatogram). The content of two extracts is similar in the polar to medium polarity region.

Table S1. ADMET profile of isolated compounds predicted by QSAR regression models

Compound	Solubility		Pharmacokinetics				Toxicity	
	ADME solubility	Solubility	Alogp98	Absorption	BBBP[a]	PPB[b]	CYP2D6 inhibition	Hepatic toxicity
1	-5.958	low	4.825	moderate	undefined	true	false	false
2	-5.938	low	4.825	moderate	undefined	true	false	false
3	-6.039	very low	3.647	good	medium	true	false	false
4	-7.145	very low	5.915	low	undefined	true	false	false
5	-7.567	very low	6.546	low	undefined	true	false	false
6	-6.442	very low	5.579	moderate	undefined	true	false	false
7	-6.21	very low	3.843	good	medium	true	false	false
8	-6.087	very low	4.333	good	undefined	true	false	true
9	-4.36	low	3.286	moderate	undefined	true	false	false
10	-4.996	low	3.974	good	undefined	true	false	false
11	-6.091	very low	5.123	good	undefined	true	false	false
12	-7.612	very low	6.447	moderate	undefined	true	false	false
13	-6.487	very low	5.48	moderate	undefined	true	false	false
14	-7.197	very low	6.212	moderate	very high	true	false	false
15	-5.293	low	4.389	moderate	undefined	true	false	false
16	-7.721	very low	7.749	very low	undefined	true	false	false
17	-7.721	very low	7.749	very low	undefined	true	false	false
Ursolic acid	-7.617	very low	6.492	moderate	undefined	true	false	false

* Discovery Studio (Dassault Systèmes Biovia Corp.) was used to predict pharmacokinetic properties based on QSAR models. [a] Blood-brain barrier penetration. [b] Plasma protein binding.
